# Psychological Distress Among Older LGBT and Non-LGBT Asian Americans: The Influence of Minority Stress

**DOI:** 10.1093/geroni/igaa057.2125

**Published:** 2020-12-16

**Authors:** Chien-Ching Li, Alicia Matthews, XinQi Dong

**Affiliations:** 1 Rush University, Chicago, Illinois, United States; 2 University of Illinois at Chicago, Chicago, Illinois, United States; 3 Rutgers University, New Brunswick, New Jersey, United States

## Abstract

Emerging data from epidemiological studies have confirmed elevated prevalence rates for mental health conditions among the lesbian, gay, bisexual and transgender (LGBT) populations. An estimated 2.8% of Asian Americans identify as LGBT and 26% of Asian LGBT are 40 years or older. This study analyzed the California Health Interview Survey to examine differences in psychological distress between LGBT and non-LGBT older Asian Americans, and further evaluated the role of discrimination in medical care and intimate violence on psychological distress. Regression results showed older LGBT Asians had a higher psychological distress score compared to non-LGBT Asians. After adjusting for discrimination or violence, this association no longer existed. Experiencing discrimination in medical care and intimate violence were associated with higher levels of psychological stress. This study increases our knowledge of mental health among older Asian LGBT, enhancing our ability to design culturally-targeted and trauma-informed psychosocial interventions to improve outcomes in this population.

